# *Hypocalcemia* as an important differential diagnosis in patients suffering from stridor following thyroidectomy

**DOI:** 10.1002/ccr3.3559

**Published:** 2020-11-19

**Authors:** Christine Nitschke, Tarek Ghadban, Jakob Izbicki, Rainer Grotelüschen

**Affiliations:** ^1^ Department of General, Visceral and Thoracic Surgery University Medical Center Hamburg‐Eppendorf Hamburg Germany

**Keywords:** acute medicine, endocrinology and metabolic disorders, general surgery

## Abstract

It is essential to consider hypocalcemia as a cause of stridor, especially following postoperative thyroidectomy, as hypocalcemia secondary to hypoparathyroidism is an important differential diagnosis. Advances in intraoperative technology to optimize the vascularization of the parathyroid glands can help to predict and prevent patients from a postoperative hypoparathyroidism.

## Graphical abstract

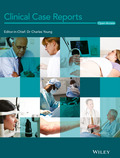

## 1 INTRODUCTION

We present the case of an adult patient with stridor following thyroidectomy. Hypocalcemia is an important differential diagnosis, as the patient was suffering from laryngospasm due to hypocalcemia secondary to postoperative hypoparathyroidism. Advances in intraoperative technology to optimize the vascularization of the parathyroid gland can help to predict postoperative hypoparathyroidism.

A newly developed stridor following total thyroidectomy in adult patients is most commonly caused by swellings, hematoma, or paralysis of the vocal cord. Although being a rare cause of stridor, laryngospasm caused by hypocalcemia due to postoperative hypoparathyroidism should also be considered as a differential diagnosis.^1^

We report an adult patient suffering from dyspnea, laryngospasm, and laryngeal stridor after undergoing a total thyroidectomy. Postoperative hypocalcemia was found to be the cause of the symptoms, which could completely be resolved after calcium replacement therapy.

## 2 CASE REPORT/CASE PRESENTATION

An adult patient underwent a total thyroidectomy for struma multinodosa at our hospital. During surgery, one parathyroid gland has been re‐implanted into the left lateral throat muscle.

On the first postoperative day, the patient complained about general discomfort, paresthesia, nausea, and dizziness at mobilization. The calcium blood level was 1.73 mmol/L, and parathyroid hormone was 17.4 pg/mL. We initiated a calcium substitution with 1 g calcium and 0.5 µg Vitamin D per day, under which the paresthesia reduced.

On the second postoperative day, the patient suddenly suffered from severe paresthesia at the entire body with dyspnea and stridor—and subjectively described a laryngospasm.

### 2.1 Investigations

Besides the laryngeal stridor, a positive Trousseau sign was noted within our clinical examination. The postoperative laboratory results showed a decrease of the calcium blood levels with a hypocalcemia of 1.68 mmol/L. The blood oxygenation level was 94% with 2l of oxygen flow, the blood pressure 160/85 mm Hg and the pulse 75/min.

A postoperative laryngoscopy had previously shown a good function of both vocal cords. Furthermore, a postoperative hematoma could be excluded by using sonography, and there was no suggestion of allergic reactions. A hypocalcemia due to postoperative primary hypoparathyroidism therefore seemed to be the explanation for the patient's laryngospasm and stridor.

### 2.2 Treatment

Considering hypocalcemia the cause of stridor and laryngospasm, an intravenous infusion of 20 mL calcium gluconate 10% (which is equivalent to 184 mg calcium) was immediately given within four hours, while electrolyte blood levels and the patient were monitored.

### 2.3 Outcome and follow‐up

The intravenous calcium substitution with 20 mL calcium gluconate 10% completely reversed all symptoms: the laryngospasm and stridor disappeared, the pulmonary auscultation was normal and the blood oxygenation level reached 99% without oxygen substitution. Additionally, the nausea and dizziness, which were present since the first operative day, disappeared after calcium substitution. A control of calcium blood levels showed an increase of calcium to 1.91mmol/l. With an oral substitution of 1.2 g calcium and 0.5 µg vitamin D per day, the patient felt more comfortable and was then discharged the following day without any severe symptoms. In the ambulant sector, the calcium blood levels and parathyroid hormone were frequently controlled by the patient's general practitioner for an optimal individualized calcium substitution, which was continued with a 1 g calcium and 1 µg vitamin D per day for more than one year after surgery.

## 3 DISCUSSION/CONCLUSION

Although laryngospasm is a rare condition after thyroidectomy, it is a serious complication of hypocalcemia and potentially lethal.^1^, ^2^ Therefore, hypocalcemia should always be considered as a differential diagnosis in adults with acute dyspnea and stridor. Hypocalcemia is the most well‐known barrier to short‐stay thyroidectomy, as patients are often discharged within less than 72 hours after surgery.^3^ Especially in the context of a primary hypoparathyroidism with insufficient parathyroid hormone secretion after thyroidectomy, we need to consider hypocalcemia besides other postsurgical complications as a cause of stridor.^4^ A prompt treatment with intravenous calcium gluconate alongside monitored magnesium levels is warranted and can resolve all symptoms.^1^, ^5^, ^6^

In most cases, symptoms of postsurgical hypocalcemia are present immediately up to 96 hours after surgery.^6^, ^7^, ^8^ Nevertheless, even years after thyroidectomy stridor can be caused by postoperative chronical primary hypoparathyroidism and a consecutive hypocalcemia.^5^ We should therefore always be aware of hypocalcemia in patients who present stridor at any time‐point after thyroidectomy.

Exclusion of other possible causes of stridor including laryngoscopy and sonography is essential in the postoperative setting. Main postoperative complications of thyroidectomy are injuries to the parathyroid glands and to the laryngeal nerves, but also tracheal compression secondary to hematoma or tracheomalacia.^5^, ^6^, ^9^, ^10^ Another possible differential diagnosis of dyspnea is a pulmonary edema due to volume overload, which can be caused by a membranous nephropathy linked to hypothyroidsm.^11^ An injury of the parathyroid glands can lead to the manifestation of temporary and even permanent hypocalcemia due to low parathyroid hormone levels.^10^ It occurs in up to 15% of postoperative patients, who can be symptomatic or asymptomatic.^5^, ^10^, ^12^ Especially at risk are patients who already present low levels of parathyroid hormone at surgery and in who the parathyroid glands are resected.^10^ Hypocalcemia causes an increased neuromuscular irritability that can lead to muscular cramps, circumoral numbness, and paresthesia of the feet and hands—or even to medical emergencies such as laryngospasm, myocardial dysfunction, seizures, generalized or focal tonic muscle cramps in severe cases.^4^, ^5^, ^13^ Laryngeal stridor that progresses to laryngospasm can be one of the first signs of hypocalcaemic tetany.^6^

New advances in technology and microvascular surgical techniques allow the prediction of postoperative hypoparathyroidsm during surgery and can be future approaches for preventing patients from postoperative hypoparathyroidsm: Indocyanine green fluorescence angiography can be used to score the perfusion of the parathyroid glands during surgery and microvascular operative techniques used to preserve the blood supply of each parathyroid gland.^14^, ^15^

In conclusion, we would like to emphasize that it is essential to consider hypocalcemia as a cause of stridor. Especially in postoperative stridor after thyroidectomy, hypocalcemia secondary to hypoparathyroidism is an important differential diagnosis.^6^, ^10^ In particular, patients with a re‐implantation of a parathyroid gland should be monitored closely and advances in technology can help to predict and prevent patients from a postoperative hypoparathyroidism.

## CONFLICT OF INTEREST

None declared.

## AUTHOR CONTRIBUTIONS

CN is the corresponding author, who wrote the case report and was involved in the patient's postoperative treatment. TG wrote and reviewed the case report and was involved in the patient's treatment as a supervisor of endocrine surgery. JI wrote and reviewed the case report as the chief of surgery, who was involved in the patient's treatment. RG took part in writing and finalizing the case report and was involved in the patient's surgery and treatment as a supervisor of endocrine surgery.

## STATEMENT OF ETHICS

The authors affirm that the study was done in accordance with the World Medical Association Declaration of Helsinki. The patient has given written informed consent prior to the study to use the clinical data for research purposes and in particular for this case report.

## Data Availability

The data that support the findings of this study are available on request from the corresponding author. The data are not publicly available due to privacy or ethical restrictions.
